# Quantitative assessment of brachial plexus MRI for the diagnosis of chronic inflammatory neuropathies

**DOI:** 10.1007/s00415-020-10232-8

**Published:** 2020-09-23

**Authors:** Marieke H. J. van Rosmalen, H. Stephan Goedee, Anouk van der Gijp, Theo D. Witkamp, Ruben P. A. van Eijk, Fay-Lynn Asselman, Leonard H. van den Berg, Stefano Mandija, Martijn Froeling, Jeroen Hendrikse, W. Ludo van der Pol

**Affiliations:** 1 Department of Neurology and Neurosurgery, University Medical Center Utrecht Brain Center, Utrecht, The Netherlands; 2 Department of Radiology, University Medical Center Utrecht, Utrecht, The Netherlands; 3 Biostatistics and Research Support, Julius Centre for Healthy Sciences and Primary Care, University Medical Centre Utrecht, Utrecht, The Netherlands; 4 Department of Radiotherapy, University Medical Center Utrecht, Utrecht, The Netherlands; 5 Computational Imaging Group for MR Diagnostic and Therapy, University Medical Center Utrecht, Utrecht, The Netherlands

**Keywords:** Magnetic resonance imaging, Brachial plexus, Chronic inflammatory demyelinating polyneuropathy, Multifocal motor neuropathy, Diagnostic value

## Abstract

**Objective:**

This study aimed at developing a quantitative approach to assess abnormalities on MRI of the brachial plexus and the cervical roots in patients with chronic inflammatory demyelinating polyneuropathy (CIDP) and multifocal motor neuropathy (MMN) and to evaluate interrater reliability and its diagnostic value.

**Methods:**

We performed a cross-sectional study in 50 patients with CIDP, 31 with MMN and 42 disease controls. We systematically measured cervical nerve root sizes on MRI bilaterally (*C*5, *C*6, *C*7) in the coronal [diameter (mm)] and sagittal planes [area (mm^2^)], next to the ganglion (*G*_0_) and 1 cm distal from the ganglion (*G*_1_). We determined their diagnostic value using a multivariate binary logistic model and ROC analysis. In addition, we evaluated intra- and interrater reliability.

**Results:**

Nerve root size was larger in patients with CIDP and MMN compared to controls at all predetermined anatomical sites. We found that nerve root diameters in the coronal plane had optimal reliability (intrarater ICC 0.55–0.87; interrater ICC 0.65–0.90). AUC was 0.78 (95% CI 0.69–0.87) for measurements at *G*_0_ and 0.81 (95% CI 0.72–0.91) for measurements at *G*_1_. Importantly, our quantitative assessment of brachial plexus MRI identified an additional 10% of patients that showed response to treatment, but were missed by nerve conduction (NCS) and nerve ultrasound studies.

**Conclusion:**

Our study showed that a quantitative assessment of brachial plexus MRI is reliable. MRI can serve as an important additional diagnostic tool to identify treatment-responsive patients, complementary to NCS and nerve ultrasound.

**Electronic supplementary material:**

The online version of this article (10.1007/s00415-020-10232-8) contains supplementary material, which is available to authorized users.

## Introduction

Chronic inflammatory demyelinating polyneuropathy (CIDP) and multifocal motor neuropathy (MMN) are rare disorders that often respond to treatment. Diagnostic criteria have been developed to distinguish CIDP and MMN from more common neuropathies and motor neuron disorders that rely on sets of typical clinical combined with specific electrodiagnostic features [1, 2]. Diagnosing CIDP or MMN remains challenging when nerve conduction studies (NCS) do not meet the required electrodiagnostic criteria [2, 3].

Nerve imaging by means of qualitative MRI is recommended in diagnostic guidelines for cases without NCS abnormalities. MRI of the brachial plexus and cervical nerve roots shows nerve root thickening and increased T2 signal intensity in 45–57% of patients [4–7]. These abnormalities have therefore been included as a supportive criterium in the diagnostic criteria for CIDP and MMN [1, 2]. However, qualitative assessments showed low interrater reliability [8, 9]. In contrast, a quantitative assessment of nerve ultrasound showed excellent test characteristics for the detection of inflammatory neuropathies [10–13]. This suggests that quantification of MRI abnormalities may improve its diagnostic value.

Therefore, the aim of our study was to systematically assess nerve root sizes on MRI of the brachial plexus and cervical nerve roots in a large cohort of patients with chronic inflammatory neuropathies and relevant disease controls. Using these data, we investigated interrater reliability and the diagnostic value of MRI in addition to NCS and nerve ultrasound.

## Methods

### Study design

We performed a cross-sectional study in prevalent and incident patients with CIDP and MMN, and clinically relevant controls [i.e. amyotrophic lateral sclerosis (ALS) or progressive muscular atrophy (PMA)]. We used a standardized protocol to systematically assess cervical nerve root sizes, determined their diagnostic value and reproducibility and developed a risk chart including objective cut-off values for abnormality.

### Patients and clinical data

All prevalent and incident patients with an established diagnosis of CIDP or MMN, visiting our neuromuscular outpatient clinic at the University Medical Center Utrecht (UMCU), were eligible for inclusion. We used previously published diagnostic criteria for CIDP and MMN, in short for CIDP we used the diagnostic criteria as defined in the EFNS/PNS guideline and for MMN we used the Utrecht criteria [1, 2]. As disease controls, we enrolled a random sample of patients with motor neuron disease (ALS and PMA), according to the Brooks criteria [14]. We excluded patients aged < 18 years, patients with motor neuron disease that had a bulbar onset of symptoms and patients who were physically unable to undergo MRI or who met one of the routine contraindications to MRI (e.g. pacemaker, non-MRI approved surgical clips or implants, claustrophobia, a recent prosthetic operation).

We obtained demographic and clinical data, including treatment response and results from routine diagnostic work-up, i.e. diagnostic NCS and nerve ultrasound results. Treatment response was evaluated based on the discretion of the treating physician. Written informed consent was obtained from all study participants.

### Routine diagnostic work-up

#### Nerve conduction studies

Diagnostic NCS were performed using a Nicolet Viking IV EMG machine (CareFusion Japan, Tokyo, Japan) following previously described protocols [10, 15]. The results were interpreted using the EFNS/PNS criteria for CIDP (definite, probable, possible) and the Utrecht criteria for MMN (definite motor conduction block, probable motor conduction block, slowing of conduction compatible with demyelination) [1, 2].

#### Nerve ultrasound

Diagnostic nerve ultrasound was performed using a Philips Affinity 70G (Philips Medical Instruments, eL 1–48 MHz linear array transducer) following a previously published protocol [10]. In short, we collected nerve sizes of the median nerves (forearm and upper arm) and brachial plexus trunks bilaterally. We used the ellipse tool to measure cross sectional area (mm^2^) and we used cut-off values for abnormal nerve size to identify patients with a chronic inflammatory neuropathy (median nerve forearm > 10 mm^2^ and upper arm > 13 mm^2^; plexus trunks > 9 mm^2^). Nerve ultrasound was considered abnormal if nerve enlargement was present at ≥ 1 measured sites.

### Equipment and MRI parameters

All patients underwent an MRI scan of the brachial plexus and cervical nerve roots on a 3.0 T MRI scanner (Philips Healthcare, Best, the Netherlands) using a 24-channel head neck coil. All participants were positioned in supine position. We performed 3D turbo spin echo spectral presaturation with inversion recovery (SPIR) in a coronal and sagittal slice orientation with the following acquisition parameters: field of view = 336 × 336 × 170 mm, matrix size = 224 × 223, voxel size = 0.75 × 0.75 × 1 mm^3^, echo time = 206 ms, repetition time = 2200 ms, turbo spin echo factor = 76, sense factor = 3 (P reduction right/left) and 1.5 (S reduction anterior/posterior), acquisition time = 03:59 min. A coronal slab maximum intensity projection (MIP) was created as a post-processing step (slab thickness = 10 mm, number of slabs = 75).

### Nerve root measurements on MRI data

**Fig. 1:**
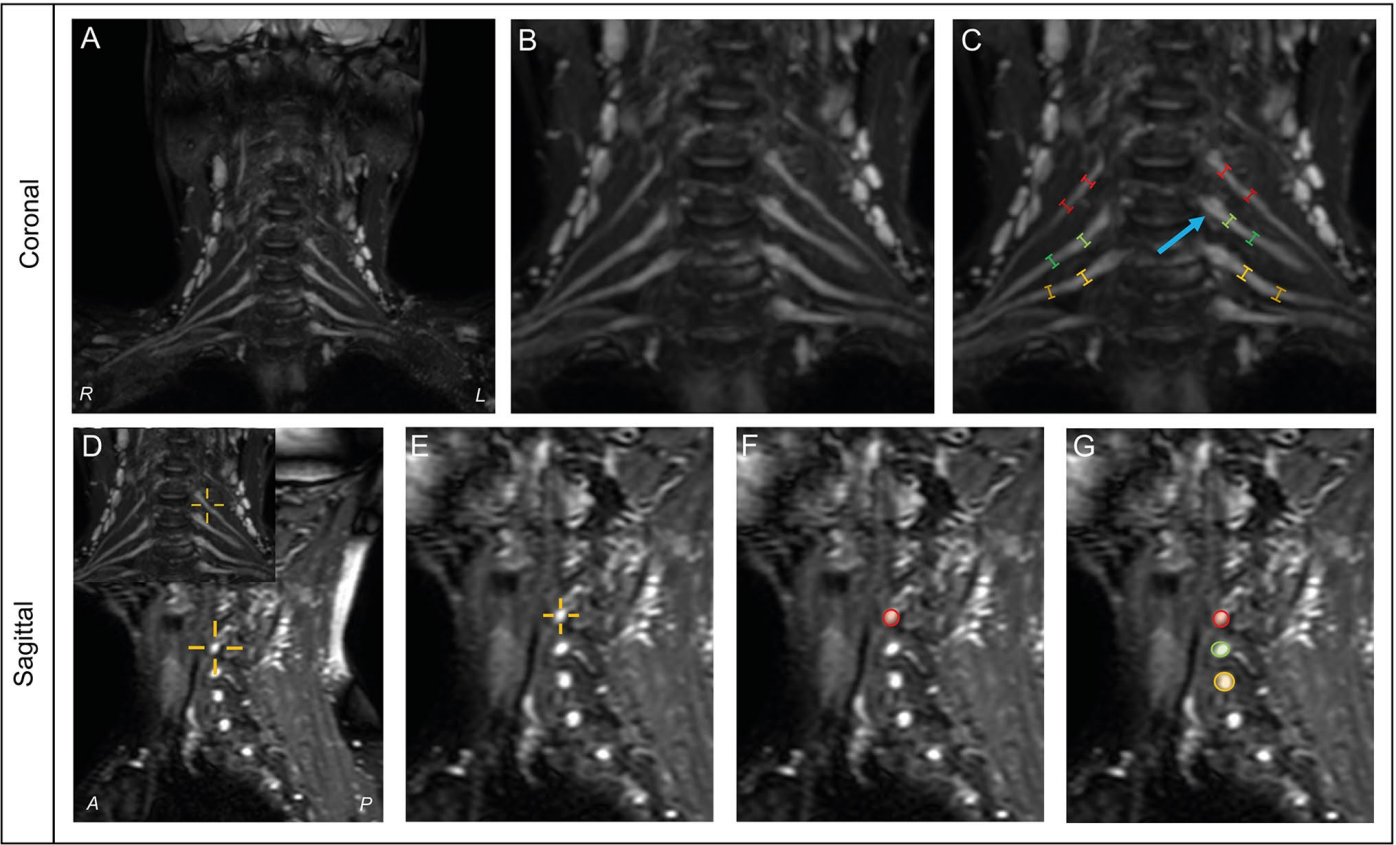
Example of nerve root measurements in coronal and sagittal planes. Method of measurements in coronal (upper) and sagittal (lower) planes. Coronal measurements in maximum intensity projection images (**a**) using 1 × zoom (**b**) and calipers placed in nerve root C5 (red), C6 (green) and C7 (yellow) next to the ganglion (blue arrow) and 1 cm distal of the ganglion (**c**). Sagittal measurements in T2 weighted fat-suppressed images using a cross-cursor to identify corresponding measurement sites (**d**) and 1 × zoom (**e**). Measurements were then performed at these corresponding measurement sites (**f**, **g**). *R* right; *L* left; *A *anterior; *P *posterior

We measured cervical nerve root sizes in coronal and sagittal planes, using PACS IDS7 21.1.2 (Sectra AB, Linköping, Sweden). We used the distance tool to measure diameters (mm) of nerve roots in coronal MIP images. Nerve root diameter was measured perpendicular on the center lines of the nerve roots, bilaterally in root C5, C6 and C7 at two predetermined anatomical sites: directly next to the ganglion (*G*_0_) and 1 cm distal from the ganglion (*G*_1_). In addition, we used the cross-cursor tool to identify the corresponding sites of these measurements on the sagittal 3D TSE SPIR, and measured cross sectional area (mm^2^) in the sagittal plane using the area tool, which is a manual tracer, resulting in 24 measurements in total per subject (duration 3–5 min per subject, Fig. 1). Zoom magnification was standardized to 1 × for all images. As anatomic variability in the brachial plexus is common and may be even more present in more distal parts [16], we decided to not perform measurements when individual nerve roots merged, divided or showed other anatomical variances. We also did not perform measurements when image quality was poor. To determine intrarater reliability, one rater (MVR) performed all measurements twice in two sessions with an interval of 1 month between the first and second sessions. To determine interrater reliability a second rater (AG) scored a random sample of 20 MRI scans from our data set. Both raters were blinded to clinical status.

### Statistical analysis

IBM SPSS Statistics (Version 25, Chicago, Illinois, United States) was used for statistical analysis. To compare patient characteristics between cases and controls, we used one-way analysis of variance (ANOVA) for numerical data and *χ*^2^ test for categorical data. To evaluate the feasibility of our method, we compared numbers of successfully performed measurements between the coronal and sagittal plane and between *G*_0_ and *G*_1_ using an independent samples *t* test. To determine mean nerve root size we also used an independent samples *t* test. Results with a *p* value < 0.05 were considered significant. To evaluate intra- and interrater reliability we used the intraclass correlation coefficient (ICC). We calculated a mean ICC of the right and left sides per measurement site. We considered an ICC < 0.50 as poor reliability, 0.50–0.75 as moderate, 0.75–0.90 as good and > 0.90 as excellent reliability [17].

#### ROC analysis and development of risk chart

We used receiver operating characteristic (ROC) analysis to determine area under the curve (AUC) per nerve root (C5, C6, C7) and for two different combinations of measurement: (1) mean of all three nerve roots bilaterally next to the ganglion (3 variables) and (2) mean of all three nerve roots 1 cm distal from the ganglion (3 variables). We then used a multivariate binary logistic model for both combinations separately with measurement sites as covariates. With the results of this model we calculated the log odds for having an inflammatory neuropathy using the following equation (Eq. 1):

1, $${\log}\left( {\frac{p}{1 - p}} \right) = \beta_{0} + \beta_{1} C5 + \beta_{2} C6 + \beta_{3} C7$$

where $\beta$_0_ is the constant, *β*_1_, *β*_2_ and *β*_3_ the logistic regression coefficients of nerve roots *C*5, *C*6 and *C*7 respectively and *C*5, *C*6 and *C*7 the diameters of the nerve roots in millimetres. Subsequently, we took the inverse logit to obtain $p$, i.e. the absolute probability of having an inflammatory neuropathy, using the following equation (Eq. 2):

2 $$p = \frac{1}{{1 + e^{{ - \left( {\beta_{0} + \beta_{1} C5 + \beta_{2} C6 + \beta_{3} C7} \right)}} }}$$

To develop a risk chart, we calculated $p$ for different combinations of *C*5, *C*6 and *C*7 and for both combinations of measurement sites. Finally, we obtained a cut-off value for $p$ obtaining 95% specificity, i.e. we determined at which $p$ we considered MRI to be abnormal.

## Results

### Patients

We included a total of 123 patients (CIDP = 50, MMN = 31, disease controls = 42). Patient characteristics are summarized in Table 1. Patients with MMN were younger than patients with CIDP and disease controls (*p* < 0.001). We found no significant differences in other baseline characteristics between groups.

**Table 1:** Patient characteristics

Parameter	Inflammatory neuropathy		Motor neuron disease	Level of significance
	CIDP	MMN		
Number of patients	50	31	42	–
Age, years (SD)	63.8 (9.4)	52.5 (11.7)	63.1 (11.2)	< 0.001*
Male (%)	42 (84.0%)	29 (93.5%)	31 (73.8%)	0.083
Disease duration, months (SD)	33.6 (65.2)	61.8 (80.5)	45.4 (38.1)	0.143
Nerve conduction study				
Inconclusive (%)	14 (28.0%)	7 (22.6%)	–	
Possible (CIDP)/slowing of conduction (MMN) (%)	9 (18.0%)	3 (9.7%)	–	
Probable (%)	2 (4.0%)	3 (9.7%)	–	
Definite (%)	25 (50.0%)	18 (58.1%)	–	
Ultrasound				
Normal (%)	10 (20.0%)	6 (19.4%)	5 (11.9%)	
Abnormal (%)	35 (70.0%)	25 (80.6%)	3 (7.1%)	
Missing (%)	5 (10.0%)	0 (0.0%)	34 (81.0%)	

*Age differs significantly between patients with MMN and patients with CIDP, and between patients with MMN and disease controls

### Nerve root measurements on MRI

#### Feasibility of measuring method

Supplemental Table 1 summarizes the number of measurements per nerve root that could be performed successfully. We obtained more measurements at *G*_0_ compared to *G*_1_ (*p* < 0.001). Measurements in the coronal plane were more often successful than in the sagittal plane (*p* < 0.001). We established that this was mostly related to early merging or dividing nerve roots and the fact that images showed lower image quality more distally.

#### Intra- and interrater reliability

Table 2 shows the intraclass correlation coefficients (ICC) within and between raters. We found moderate to good intrarater reliability in both plane orientations (ICC 0.55–0.87 in coronal plane, and 0.63–0.86 in sagittal plane). We found moderate to good interrater reliability in the coronal plane (ICC 0.65–0.90) but a poor to good reliability in the sagittal plane (ICC 0.47–0.84). Overall, we found higher consistency in measurements performed in the coronal plane orientation.

**Table 2:** Reliability of nerve root measurements on brachial plexus MRI

Site	Intrarater reliability		Interrater reliability	
	Coronal	Sagittal	Coronal	Sagittal
C5				
Ganglion	0.81 (0.74–0.86)	0.69 (0.58–0.77)	0.81 (0.58–0.92)	0.52 (0.09–0.78)
1 cm	0.55 (0.41–0.67)	0.63 (0.47–0.74)	0.78 (0.51–0.91)	0.62 (0.14–0.87)
C6				
Ganglion	0.77 (0.69–0.84)	0.68 (0.58–0.77)	0.77 (0.37–0.89)	0.47 (0.04–0.75)
1 cm	0.84 (0.77–0.89)	0.83 (0.74–0.88)	0.82 (0.58–0.93)	0.79 (0.50–0.92)
C7				
Ganglion	0.78 (0.70–0.84)	0.75 (0.67–0.82)	0.65 (0.13–0.87)	0.73 (0.44–0.89)
1 cm	0.87 (0.81–0.91)	0.86 (0.79–0.91)	0.90 (0.60–0.97)	0.84 (0.35–0.96)

Intraclass correlation coefficient (ICC) with 95% confidence interval for every measurement site in coronal and sagittal planes

#### Mean nerve root size

Mean nerve root sizes are summarized in Table 3. Nerve root sizes in patients with CIDP and MMN were larger compared to disease controls, at all predetermined anatomical sites (*p* varied from < 0.001 to 0.026).

**Table 3:** Mean nerve root sizes per measurement site

Nerve root	Inflammatory neuropathy (*n* = 81)	Control (*n* = 42)	MD (95% CI)	Level of significance
Coronal				
C5				
Ganglion (SD)	3.0 (0.8)	2.5 (0.6)	0.5 (0.3–0.7)	< 0.001
1 cm (SD)	2.8 (0.9)	2.2 (0.5)	0.6 (0.3–0.8)	< 0.001
C6				
Ganglion (SD)	3.8 (0.9)	3.3 (0.6)	0.5 (0.2–0.8)	< 0.001
1 cm (SD)	3.6 (1.1)	2.9 (0.7)	0.7 (0.3–1.1)	< 0.001
C7				
Ganglion (SD)	4.0 (0.9)	3.4 (0.7)	0.7 (0.3–1.0)	< 0.001
1 cm (SD)	3.7 (1.1)	2.8 (0.6)	0.9 (0.4–1.4)	< 0.001
Sagittal				
C5				
Ganglion (SD)	21.6 (6.8)	18.5 (5.7)	3.1 (0.7–5.6)	0.013
1 cm (SD)	20.3 (7.2)	16.7 (4.4)	3.6 (1.1–6.1)	0.005
C6				
Ganglion (SD)	27.2 (9.1)	23.4 (5.2)	3.8 (0.8–6.8)	0.013
1 cm (SD)	25.3 (11.5)	19.2 (6.5)	6.1 (2.0–10.2)	0.004
C7				
Ganglion (SD)	26.4 (10.4)	22.0 (5.4)	4.4 (1.5–7.2)	0.003
1 cm (SD)	23.1 (14.7)	16.1 (4.3)	7.1 (0.9–13.3)	0.026

Nerve root sizes are mean. Coronal measurements are in millimetres (mm). Sagittal measurements are square millimetres (mm^2^)

*MD *mean difference, *CI *confidence interval, *SD *standard deviation

### ROC analysis and development of risk chart

**Fig. 2:**
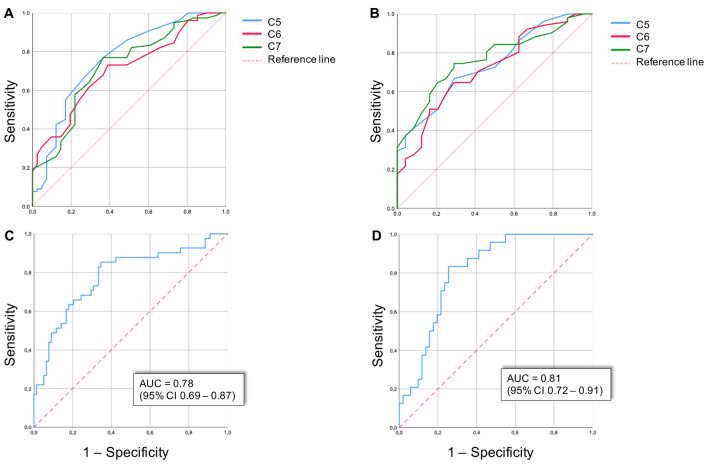
ROC analysis of nerve root size measurements on MRI. ROC curves of measurements per nerve root next to the ganglion (**a**) and 1 cm distal of the ganglion (**b**) are shown in the upper panels. Combined ROC curves of measurements next to the ganglion (**c**) and 1 cm distal of the ganglion (**d**) are shown in the lower panels. Combined measurements are expressed as area under the curve (AUC) and 95% confidence interval (CI)

**Fig. 3:**
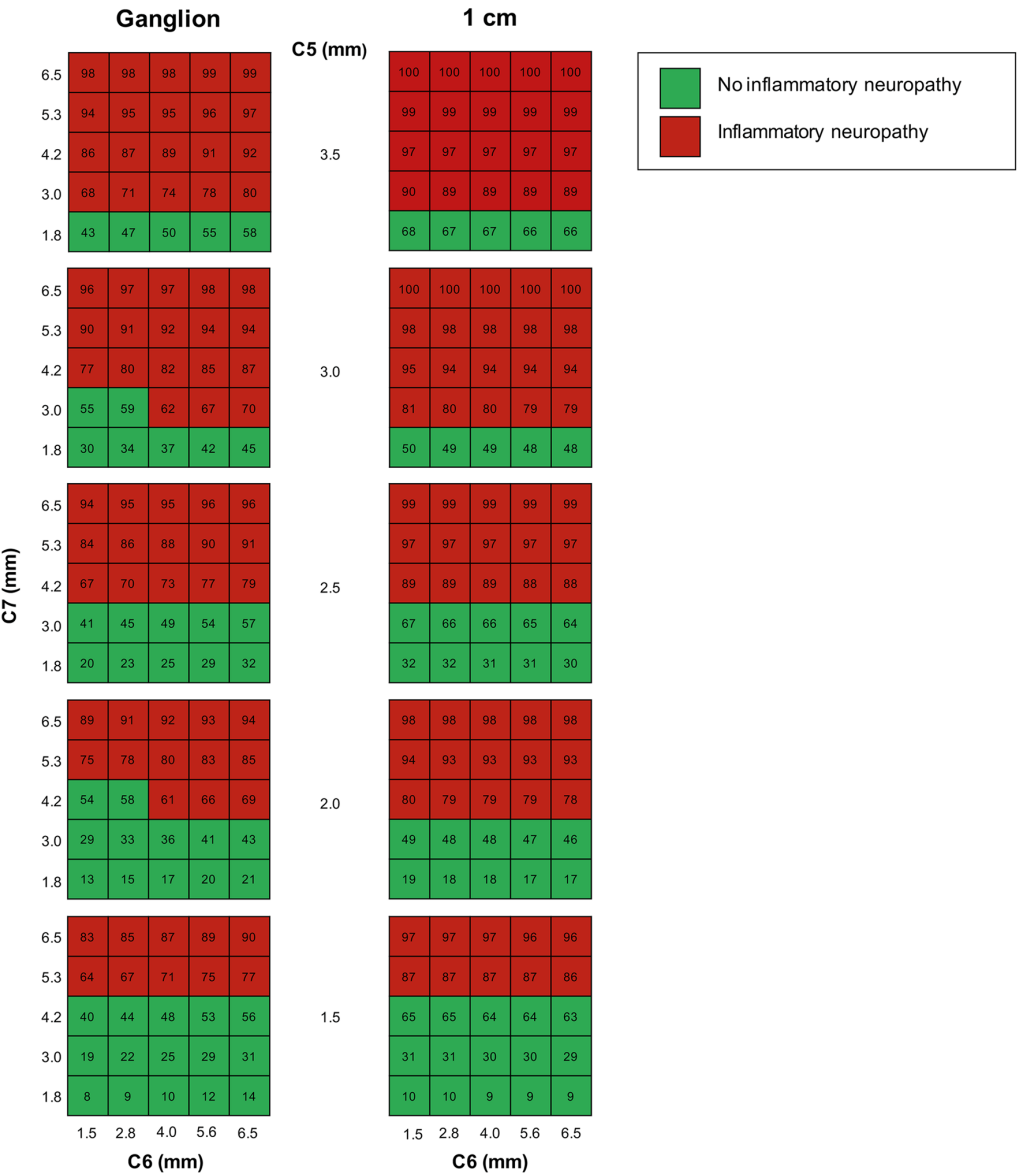
Risk chart for predicting CIDP or MMN based on nerve root sizes. Risk charts for measurements next to the ganglion (left panels) and 1 cm distal from the ganglion (right panels). The risk chart provides the absolute risk of having CIDP or MMN based on different combinations of nerve root thickness of nerve root *C*5, *C*6 and *C*7. Every cell of the table contains the probability of having CIDP or MMN (e.g. for measurements next to the ganglion (left panels): if *C*5 is 1.5 mm, *C*6 is 1.5 mm and *C*7 is 1.8 mm, the probability of having CIDP or MMN is 8%). A probability of ≥ 61% for measurements next to the ganglion and ≥ 69% for measurements 1 cm distal from the ganglion were considered abnormal (cells in red). The axes range between the 95% lowest and highest measurements

Sagittal measurements were less often successful because of lower data quality and overall lower reliability (Table 2 and supplemental Table 1). We therefore decided to exclude the measurements in the sagittal plane from further analysis. Results from the ROC analysis are shown in Fig. 2. We found a comparable AUC for both predetermined anatomical sites in the coronal plane (*G*_0_ and *G*_1_). We developed a risk chart (Fig. 3) that predicts the absolute chance of having a chronic inflammatory neuropathy, based on different combinations of nerve root sizes of *C*5, *C*6 and *C*7.

#### The added value of MRI

**Fig. 4:**
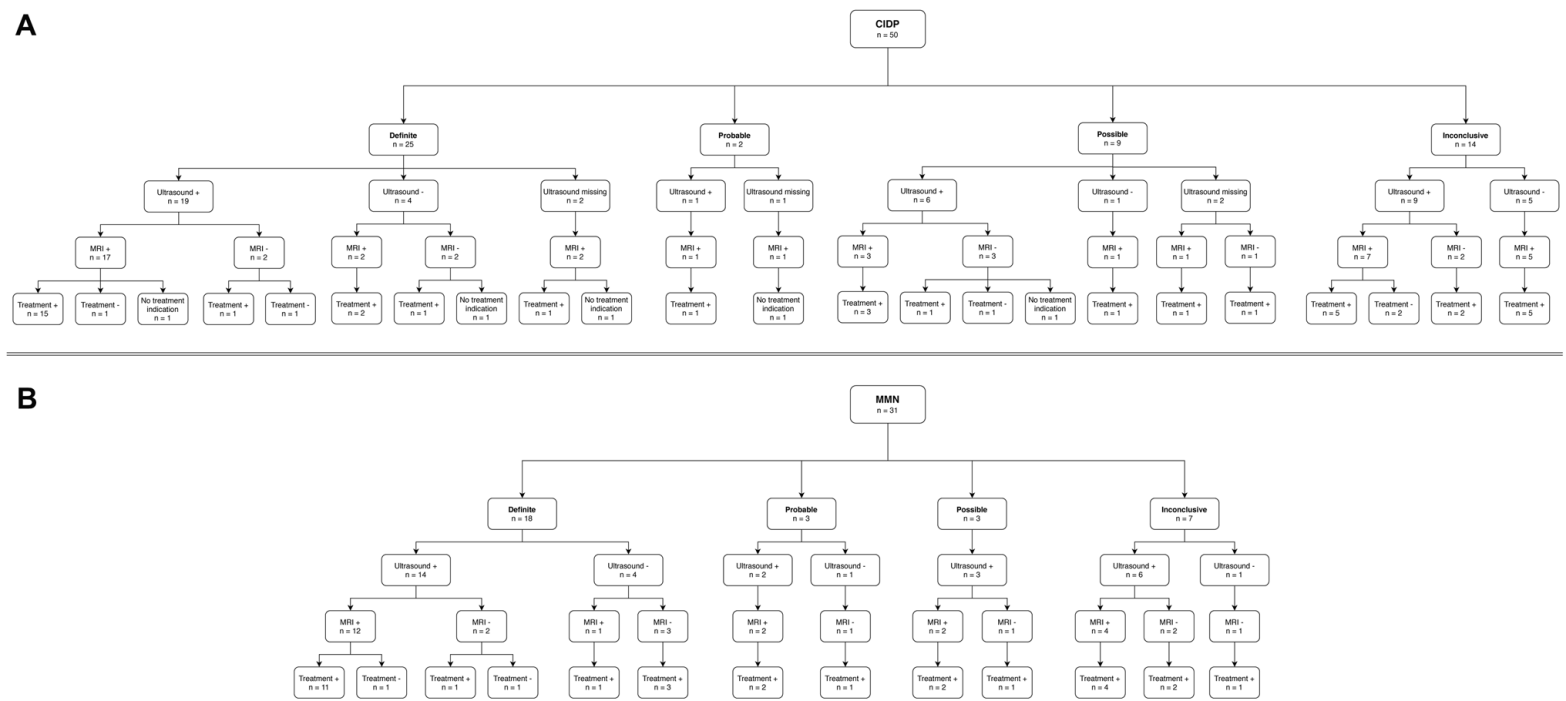
Results of NCS, ultrasound and MRI in patients with CIDP and MMN. Flow chart of CIDP patients (**a**) and MMN patients (**b**) showing outcome of nerve conduction studies, nerve ultrasound of the brachial plexus and median nerve, MRI of the brachial plexus and cervical nerve roots and treatment response

ROC analysis showed that at a set specificity of 95%, the sensitivities are 27% for *G*_0_ and 17% at *G*_1_. With this specificity, a probability of ≥ 61% for measurements at *G*_0_ and ≥ 69% at *G*_1_ in the risk chart were considered abnormal or likely to have a chronic inflammatory neuropathy (Fig. 3). With these cut-off values, we determined which patients in our data set had an abnormal MRI and we investigated the added value of brachial plexus MRI in addition to NCS and nerve ultrasound. We found that NCS combined with nerve ultrasound identified most patients with an inflammatory neuropathy. The majority of patients with abnormal ultrasound findings also had abnormal MRI findings (Fig. 4). However, 5/50 (10%) patients with CIDP had an abnormal MRI result, while NCS did not fullfill the criteria for CIDP and ultrasound did not show abnormalities. All patients had a good response to treatment. Clinical symptoms and laboratory findings of these five patients are summarized in Table 4. MRI did not have any added diagnostic value for MMN.

**Table 4:** Patient characteristics of patients with CIDP who did not fulfil diagnostics criteria on NCS and without ultrasound abnormalities

Patient	Male/female	Clinical presentation	NCS	Electrodiagnostic criteria*	Liquor protein in g/L (normal 0.00–0.40)	Treatment and dosage	Response to treatment
1	Male	Symmetrical weakness in proximal and distal arm and leg muscles; loss of vibration, touch and position sense in arms and legs; areflexia	CMAP↓ right median, right peroneal and left tibial nerve. ↑DML bilateral median nerves. ↓SNAP bilateral median, ulnar, radial and sural nerves	Not compatible	0.39	Intravenous immunoglobulins, 40 g every 3 weeks	Improvement of pinch force right hand from 55 to 100 kPa, improvement pinch force left hand of 30 kPa to 98 kPa, measured with Martin vigorimeter
2	Male	Asymmetrical weakness in proximal and distal right arm muscles and right leg muscles; loss of vibration sense distal from knees; low reflexes in the arms, areflexia in the legs	CMAP↓ bilateral median, right ulnar, bilateral peroneal and left tibial nerve. ↑DML bilateral median nerves. Normal SNAP’s	Not compatible	0.48	Intravenous immunoglobulins, 30 g every 3 weeks	Improvement of dorsal flexion of right foot, measured with myometry by physiotherapist
3	Male	Asymmetrical weakness in proximal and distal right arm; tremor; loss of vibration sense in feet; areflexia	CMAP↓ right median nerve. ↑DML left median nerve. ↓SNAP bilateral median and sural nerves	Not compatible	0.61	Intravenous immunoglobulins, 40 g every 4 weeks	Improvement of MRC 4 to 5 in right arm, measured by treating physician
4	Male	Symmetrical weakness in proximal and distal leg muscles; fluctuating pain in legs; loss of vibration sense in feet; areflexia	CMAP↓ right peroneal and left tibial nerve. ↑DML right median and left ulnar nerve. ↓SNAP bilateral sural nerves	Not compatible	0.70	Methylprednisolone 1000 mg every 4 weeks	Improvement of MRC 3 to 4 (right) and MRC 4 to 5 (left) in quadriceps muscles, improvement of MRC 0 to 4 in left anterior tibial muscle, measured by treating physician. For 3 years ago in wheel chair, currently walks an hour (with walking stick)
5	Male	Symmetrical weakness in extensor hallucis longus muscle; loss of vibration and touch sense in feet up to the knees; low reflexes in the arms, areflexia in the legs	CMAP↓ left median, bilateral ulnar and peroneal, right tibial nerve. ↑DML right median nerve. ↓SNAP bilateral median, right ulnar, left tibial, bilateral sural	Not compatible	0.42	Single therapy of intravenous immunoglobulins, 40 g during 5 days	Improvement of touch sense (currently only persistent in feet), better balance, observed by treating physician

## Discussion

Quantitative assessment of brachial plexus MRI has acceptable interrater reliability and can be used in the diagnostic workup of patients who may have an inflammatory neuropathy. It can complement NCS and nerve ultrasound for the diagnosis of CIDP, but not MMN. A quantitative assessment of MRI of the brachial plexus and cervical nerve roots with high specificity identified 10% additional patients who responded to treatment but had not been identified by NCS and nerve ultrasound.

MRI is part of the current diagnostic criteria for CIDP and MMN and is recommended in particular for the identification of elusive cases, i.e. those without clear NCS abnormalities [1, 2, 18–21]. This is based on several MRI studies that showed cervical nerve root thickening and increased signal intensity on brachial plexus MRI in a subgroup of patients with chronic inflammatory neuropathies [7, 20]. A clear limitation of qualitative assessment of brachial plexus MRI as it is used nowadays is its low interrater reliability [8, 9]. Few studies have explored the feasibility and use of a quantitative MRI assessment and only in small groups of patients and healthy controls [9, 22–25]. Estimates of the upper limit of normal for cervical nerve root size in healthy controls ranged between 4 and 5 mm. Analysis of our data from a large cohort of patients with CIDP and MMN showed that combinations of nerve root size are probably more useful than a fixed cut-off. This may be explained by the patchy nature of inflammatory changes. We found that six bilateral measurements close to the ganglion of root *C*5, *C*6 and *C*7 in coronal plane was easy to implement in routine practice (~ 3 min per subject) and resulted in optimal test characteristics with high specificity levels. Sensitivity levels of quantitative assessment of brachial plexus MRI were lower than those reported in qualitative studies [23, 24]. This may be explained by some inclusion bias in earlier studies, as shown by another recent prospective cohort study that also reported a relatively low sensitivity of qualitative brachial and lumbosacral plexus MRI in patients with suspected CIDP [21]. Importantly, test–retest reliability for quantitative measurements was good, which is supported by data from another recent study [23].

We analyzed the diagnostic value of a quantitative assessment of MRI next to NCS and nerve ultrasound studies [10, 12, 13]. MRI helped to identify patients with a clinical phenotype compatible with CIDP but who did not fulfil the diagnostic criteria of NCS and who did not have ultrasound abnormalities. In this sense, MRI complements nerve ultrasound, which has an excellent sensitivity as shown in previous studies [10, 13]. Quantitative assessment of brachial plexus MRI identified an additional 10% of patients who responded to treatment, which is clinically relevant. MRI should, therefore, be considered as an additional diagnostic tool when there is a strong clinical suspicion of CIDP, particularly when NCS and nerve ultrasound results are normal. Nerve ultrasound, and especially the required expertise, is not always available in all medical centres. In these centres MRI could be used as an additional tool to NCS and laboratory findings, although physicians should always consider the poor sensitivity of MRI when interpreting results.

Our study comprises a relatively large number of patients with MMN and CIDP, although we acknowledge that the group sizes in studies on rare neuropathies are almost always a limitation. Our control group was homogeneous and did not include a spectrum of mimics as in previous studies. This was a deliberate choice since ultrasound studies showed that it is unlikely that nerve root sizes are enlarged in patients with axonal neuropathies [10]. We also acknowledge that both nerve imaging and NCS may fail to discriminate CIDP from certain rare mimics, such as hereditary demyelinating polyneuropathies, paraproteinaemic polyneuropathies and amyloidosis. However, clinical phenotypes and laboratory findings in these rare mimics will often guide a clinician to the right diagnosis without the use of nerve imaging techniques.

We show that quantitative assessment of MRI of the brachial plexus and cervical nerve roots is a reliable and useful tool for the diagnostic workup of patients who may have a chronic inflammatory neuropathy. A quantitative approach is feasible and does not have the limitation of high interrater variability of the currently used qualitative assessments.

## Electronic supplementary material

Below is the link to the electronic supplementary material.

Supplementary file1 (DOCX 13 kb)

## Data Availability

The data that support the findings of this study will be available on request from the corresponding author.
