# Supplementary material for: Quantitative assessment of brachial plexus MRI for the diagnosis of chronic inflammatory neuropathies
Source: J Neurol. 2020 Sep 23;268(3):978–88. doi: 10.1007/s00415-020-10232-8 (PMC7914242; doi:10.1007/s00415-020-10232-8)
Supplement: Supplementary file 1 — Supplementary file1 (DOCX 13 kb) [file 415_2020_10232_MOESM1_ESM.docx]

**Supplemental table 1.** Rate of overall successful measurements per nerve root on brachial plexus MRI performed by one rater

| Nerve root | | Coronal | | Sagittal | |
| --- | --- | --- | --- | --- | --- |
|  |  | *Ganglion* | *1 cm* | *Ganglion* | *1 cm* |
| C5 | *Right* | 120 (97.5%) | 100 (81.3%) | 115 (93.5%) | 74 (60.2%) |
|  | *Left* | 119 (96.7%) | 85 (69.1%) | 110 (89.4%) | 56 (45.5%) |
| C6 | *Right* | 120 (97.5%) | 96 (78.0%) | 120 (97.5%) | 89 (72.4%) |
|  | *Left* | 122 (99.2%) | 87 (70.7%) | 118 (95.9%) | 66 (53.7%) |
| C7 | *Right* | 118 (95.6%) | 73 (59.3%) | 116 (94.3%) | 68 (55.2%) |
|  | *Left* | 119 (96.7%) | 70 (56.9%) | 114 (92.7%) | 60 (48.8%) |

Number of measurements performed per nerve root differed between coronal and sagittal plane due to lower image quality, and between measurement site (next to ganglion or 1 cm distal from ganglion) due to merging or dividing of nerve roots.
